# Debriefing in practice: a cross-sectional observational study of self-reflection competence, psychological safety and speak-up in paramedic training

**DOI:** 10.1186/s12909-026-09502-2

**Published:** 2026-05-27

**Authors:** Nele Sommer, Hartwig Marung, Reinhard Strametz, Matthias Raspe

**Affiliations:** 1 Department of Infectious Diseases, Respiratory Medicine and Critical Care Medicine, Charité –University Medical Center Berlin, corporate member of Freie Universität Berlin and Humboldt Universität zu Berlin, Charitéplatz 1, Berlin, 10117 Germany; 2 Department of Anesthesiology, Surgical Intensive Care Medicine, Emergency Medicine and Pain Therapy, Klinikum Bielefeld, Campus Mitte, Bielefeld, Germany; 3 Department of Anesthesiology and Intensive Care, University of Lübeck, Lübeck, Germany; 4 Institute of Occupational, Social and Environmental Medicine, Goethe University Frankfurt, Frankfurt am Main, Germany

**Keywords:** Debriefing, Psychological safety, Reflective practice, Speak-up behaviour, Paramedic training, Emergency medical services, Workplace-based learning

## Abstract

**Background:**

Debriefing is a central component of workplace-based learning in the training of paramedics. While their effects are well documented in simulation and hospital settings, little is known about how debriefing quality, psychological safety, speak-up behaviour, and self-reflection competence are interrelated in real-world emergency medical service settings. The aim of this study was to investigate these relationships and analyze potential differences between paramedic trainees and paramedic field instructors.

**Methods:**

A cross-sectional observational study was conducted using a standardized online survey tool (July–November 2025). The study aimed to assess psychological safety, speak-up behaviour, perceived debriefing quality, and self-reflection competence. Data were analyzed descriptively and using group-specific Pearson correlations.

**Results:**

A total of 177 participants, including 61 paramedic field instructors and 116 trainees, were analyzed. Trainees and paramedic field instructors differed significantly with regard to debriefing quality (*p* < .001) and self-reflection competence (*p* < .05), while no significant differences were observed for psychological safety or speak-up. Among paramedic field instructors, self-reflection competence correlated strongly with debriefing quality (*r* = .67; *p* < .001). Among trainees, the strongest correlation was found between psychological safety and debriefing quality (*r* = .61; *p* < .001). Psychological safety was significantly associated with utilization of speak-up in both groups.

**Conclusions:**

Debriefing should not be considered merely as retrospective discussions, but rather as learning environments that are intentionally designed. The findings suggest that debriefing quality is closely associated with reflective facilitation and a psychologically safe environment, particularly for trainees. The training of on-the-job instructors should therefore specifically address, in addition to methodological facilitation skills, the design of interactions that promote reflection and foster a sense of safety, in order to sustainably improve learning, speak-up culture, and team communication.

**Trial registration:**

This study is registered in „Deutsches Register Klinischer Studien“ (DRKS-ID: DRKS00037257, Registration Date: 03.07.2025).

## Introduction

Debriefing plays a key role in paramedic field training: As a facilitated team reflection, they draw on real-life field experiences and enable participants to link clinical practice, team processes, and decision-making logic based on specific cases and experiences, thereby facilitating the transfer of learning [1–3]. Debriefing is therefore a key component of experiential learning (“reflective practice”) and the development of professional competence - even beyond formal training [4]. In the present study, the term “debriefing” refers specifically to hot debriefing conducted in close temporal proximity to real-world emergency medical events [1].

There is consistent evidence from both clinical and simulation-based settings that debriefing has positive effects, including specific aspects like teamwork and performance [5]. Meta-analyses report improvements in team performance and show that structured support can enhance the perceived quality and usefulness of debriefing [2, 6]. At the same time, challenges concerning implementation are described. These include, in particular, inadequately trained facilitators and a lack of supporting infrastructure [2, 4, 7].

A recurring issue in debriefing research is that the quality of debriefing is rarely systematically assessed as a potential influencing factor or taken into account in analyses. Particularly in real-world settings outside of clinical environments, variability in how debriefing is structured can be expected, as previous research has pointed to a lack of trained facilitators (e.g [4]).Consequently, the way debriefing is structured may influence learning effectiveness and safety culture by promoting psychological safety and enhancing speak-up.

A key prerequisite for effective debriefing is a sense of psychological safety within the team: an environment in which errors, questions, and uncertainties can be openly addressed, e.g. by voicing concerns or acknowledging gaps in knowledge without fearing negative consequences [8, 9]. Psychological safety is associated with team learning and team performance in healthcare teams, and promotes speak-up as a proactive and constructive challenge to the status quo [10–12].

When applied to emergency medical services, psychological safety is particularly challenging due to frequently changing team compositions, time pressure, and limited prior collaboration between team members. It is therefore plausible that trainees, in particular, face an increased risk of experiencing lower psychological safety - with potential consequences for their learning processes and reflection during debriefing [11].

The main objective of debriefing is to reflect one’s own actions and those of the group. Accordingly, self-reflection competence becomes an important individual resource. A process-oriented model of self-reflection that also takes volitional aspects into account was developed by Berger [13] and comprises six sub-areas (including willingness to reflect, cause analysis, goal setting, and success in reflection). Since this process logic closely resembles the typical phases of a hot debriefing, it can be assumed that the quality of the debriefing (particularly effective facilitation) and psychological safety jointly influence the depth of reflection that is actually achieved. Hot debriefing follow a certain structure: Capturing emotions and initial impressions, a brief summary from different perspectives, analysis of successful aspects, analysis of areas for improvement, and finally the formulation of concrete learning points and/or tasks arising from the discussion (e.g., skills training, feedback to quality management, …) [1, 14].

However, little is known about how these factors (a) psychological safety and speak-up, (b) self-reflection competence, and (c) perceived debriefing quality interact in real-world German emergency medical services settings,, and whether these relationships differ between trainees and paramedic field instructors.

### Objective

The objective of this study is to gain a better understanding of debriefing processes in real-world, non-clinical emergency medical settings and to identify areas for improvement and training. The study examines the interaction between (a) psychological safety and speak-up, (b) self-reflection competence, and (c) subjectively perceived debriefing quality. This combination of factors has rarely been studied in real-world debriefing within emergency medical services. Based on the existing literature, positive correlations between the individual factors can be inferred.

### Methods

### Study design

We conducted a cross-sectional observational study using a standardized online questionnaire. The aim was to provide a descriptive analysis and to examine associations (correlations) between psychological safety, speak-up behaviour, self-reflection competence, and perceived debriefing quality in a real-world, non-clinical emergency medical setting.

### Setting and recruitment

Data were collected between July and November 2025 from [1] the fire departments in the cities of Hamburg and Berlin via their affiliated vocational schools, and [2] nationwide via the distribution lists of the German Society for Rescue Sciences (DGRe) and by reaching out to additional vocational schools. The study link was distributed via the respective institutions. Participation was voluntary and anonymous. Reminders to participate were sent out every three weeks.

Both participating departments operate within the German emergency medical services system and follow the nationally standardized training regulations defined by the Paramedic Training and Examination Ordinance [15], which governs both training content and the qualifications required for paramedic field instructors. While organizational procedures (e.g., standard operating procedures) are locally defined, core structures and roles are comparable across institutions. Work environments within professional fire departments are typically characterized by close team interaction, although comparatively more hierarchical structures may be present compared to other emergency medical services providers.

### Participants: inclusion and exclusion criteria

The study included individuals aged 18 or older who were either (a) trainees in the standard three-year paramedic training program in Germany or (b) practicing paramedic field instructors in the emergency medical services. Trainees in training programs with individual content (e.g., specific programs of individual professional fire departments) were excluded. Inclusion and exclusion criteria were assessed at the beginning of the survey.

### Sample size calculation

The sample size calculation was based on the primary analysis of correlations within each group (trainees and paramedic field instructors). A target sample size of *n* = 85 per group was defined a priori to detect associations of at least moderate strength (*r* = .30) with α = 0.05 and a power of 0.80. Accordingly, the study was specifically powered to examine within-group relationships rather than between-group differences. The choice of a minimum detectable effect size of *r* = .30 was based on Cohen’s conventions [16] and reflects a realistic assumption for associations between complex psychological and social constructs. In line with recommendations from the social sciences, this threshold was defined a priori as a practically meaningful effect in the absence of prior empirical estimates [17].

### Survey instruments and variables

The survey instruments for the variables psychological safety, speak-up, self-reflection competence, and debriefing quality are presented in Table 1. Additional contextual variables potentially relevant to debriefing experiences were also assessed.

**Table 1:** Survey instruments and potential confounders

Variable and instrument	Number of Items	Scale	Interpretation
Psychological safety (Psy-Safety-Check) (9, 18)	7	7-point Likert scale	Higher scores = stronger psychological safety
Speak-up (Speak-Up-Check) (19)	4	5-point Likert scale	Higher scores = stronger speak-up behaviour Speak-up was interpreted as a behavioural aspect of team communication and as an indicator of psychological safety
Debriefing quality (adapted elements from the Debriefing Assessment for Simulation in Health Care (DASH) (20) The adaptation was limited to linguistic adjustments (e.g., pronouns/setting); (Subscales: Effective learning atmosphere, Structure, Reflective discussion, Analysis of Action, Future Orientation)	23	7-point Likert scale, with the additional response option “not observed”	Higher scores = higher perceived quality of the debriefing
Self-reflection competence according to Berger (2021) (13) (Subscales: Willingness to reflect, Descriptive reflection, Cause analysis, Goal setting, Action optimization, Success of reflection)	42	7-point Likert scale	Higher scores = more developed self-reflection competence
Potential confounders (familiarity within the team, self-reported prior knowledge/training in the areas of CRM, human factors, debriefing, reflective practice, year of training [trainees] or professional experience in emergency medical services [paramedic field instructors])		categorical	

For all variables, previously validated measurement instruments were used: Psy-Safety-Check [18], Speak-Up-Check [19], self-reflection competence - questionnaire [13]. The assessment of debriefing quality was based on items derived from the Debriefing Assessment for Simulation in Healthcare (DASH). The DASH is originally designed as an observational rating tool focusing on clearly defined and observable facilitator behaviours during debriefings [20]. As the items are based on observable facilitator behaviours, they were considered suitable for capturing participants’ subjective perceptions of debriefing quality in a self-report format. In addition to its use as a rater-based instrument, the DASH has also been applied in participant-based (e.g., DASH Student Version [21]) and self-assessment formats (e.g., Instructor Version [22]), allowing evaluations of debriefing practices from different perspectives.

The adaptation was limited to minor linguistic modifications, specifically the adjustment of pronouns to ensure gender-inclusive language (e.g. his/her). In addition, subscale Element 1 was removed, as it is specifically tailored to simulation settings. A response option (“not observed”) was added to account for situations in which specific behaviours could not be assessed by participants. No further modifications to item content, scaling, or structure were made.

While the DASH has demonstrated acceptable psychometric properties, particularly in structured simulation-based settings [20], it was originally developed as an observational assessment tool. Previous studies have indicated that its psychometric properties may vary depending on the context and mode of application [23, 24].The transfer to a self-report format may influence its measurement properties and thus represents a potential limitation of the study.

To examine the psychometric properties of the adapted version of DASH, internal consistency and item characteristics were analyzed using the present sample.

All analyses were conducted separately for trainees and instructors to account for potential differences in response patterns between groups. Reliability analyses were based on complete cases (i.e., without imputation of missing values), resulting in varying sample sizes across analyses. Cronbach’s α values of ≥ 0.70 were considered acceptable, ≥ 0.80 good, and ≥ 0.90 excellent. Corrected item–total correlations and item–subscale correlations of ≥ 0.30 were interpreted as indicating adequate (≥ 0.30) and high (≥ 0.50) item discrimination [17, 25]. These analyses were used to explore internal consistency, item homogeneity, and the internal structure of the instrument, including potential overlap between subdimensions.

As DASH was originally developed as an observational rating tool for simulation-based settings and was adapted in this study for self-report use in a real-world emergency medical context, analyses were considered exploratory. No assumptions were made regarding the transferability of the original factor structure. In particular, the dimensional structure of the original instrument was not assumed to be preserved in this context.

### Data preparation

Total scores (and, where relevant, subscale scores) were calculated according to the respective scales; higher scores reflect higher levels of the construct. Questionnaires were excluded if they were fully not completed; another was excluded because it was not possible to assign the respondent to either the group of paramedic field instructors or trainees.

For individual missing item values within a scale, scale scores were calculated as the means of available items for each participant. Consequently, the number of observations varied slightly across analyses due to variable-specific missingness. To assess the robustness of the findings, sensitivity analyses using multiple imputations were conducted. Results based on multiply imputed data were comparable to those obtained from the original dataset, indicating that the findings were robust to the handling of missing data.

### Statistics

The statistical analyses were performed using IBM SPSS (Version 31). Descriptive statistics are reported as means, standard deviations, and ranges. Because the study focused primarily on associations within groups, Pearson correlations were calculated separately for trainees and field instructors. Group differences in scale scores were explored using Mann–Whitney U tests. Statistical significance was set at *p* < .05 (two-tailed). In addition, correlations for relevant subscales were evaluated exploratively. Correlation coefficients in the range of 0.1–0.3 can be interpreted as weak, those from 0.3 to 0.5 as moderate, and those in the range of 0.5–0.7 as strong [26].

Internal consistency was assessed using Cronbach’s α for the overall scale and for each subscale. Inter-item correlations as well as corrected item–total and item–subscale correlations were calculated to evaluate item homogeneity and the internal structure of the instrument [27, 28].

Given the exploratory nature of the study and the primary focus on examining bivariate associations within groups, no multivariable adjustment models were specified a priori. In addition, the sample size in relation to the number of variables assessed, multivariable models would have been at risk of overfitting and unstable parameter estimates. Potential contextual variables were therefore analyzed exploratively to identify possible associations with the main constructs.

## Results

**Fig. 1:**
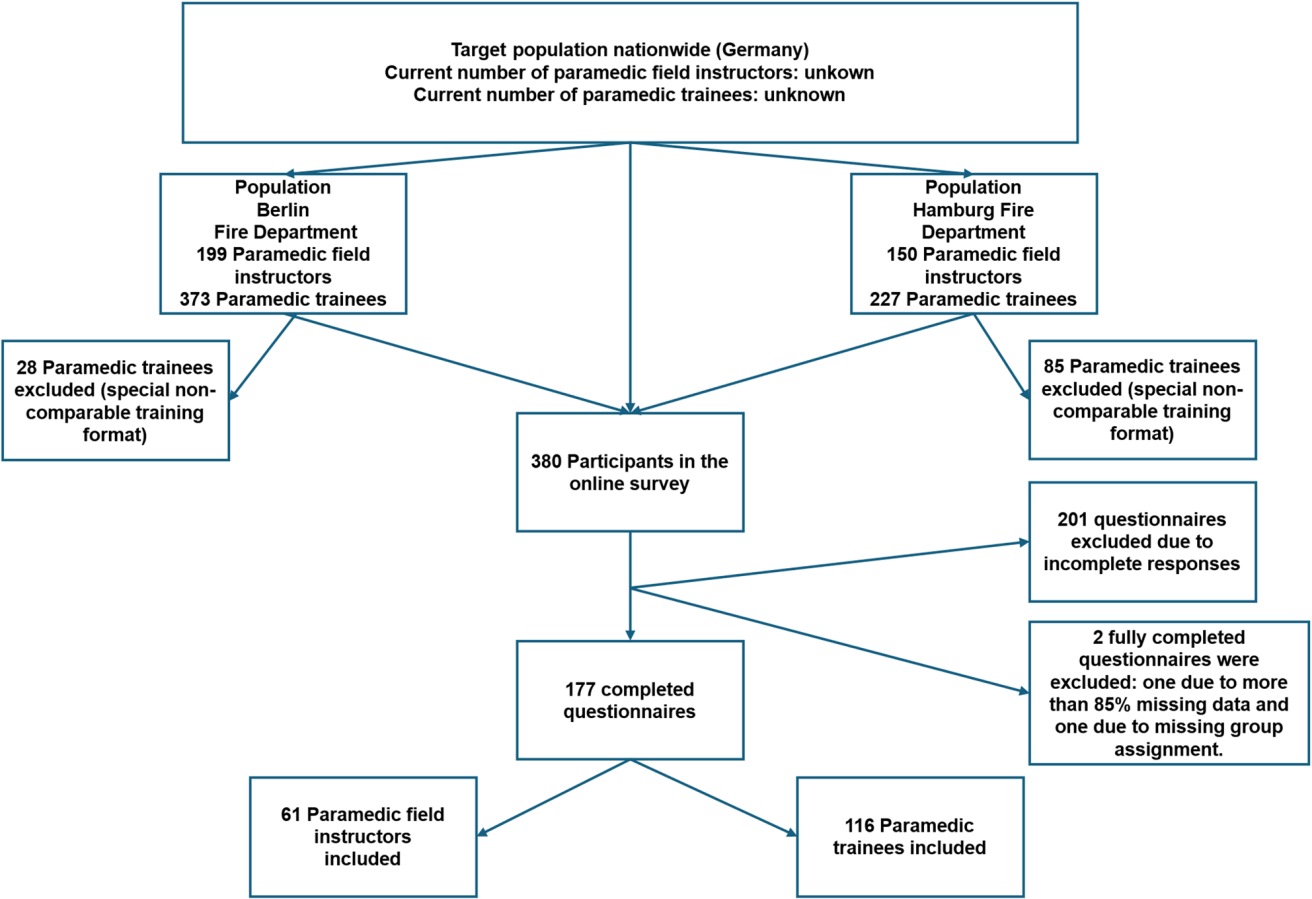
Flowchart of study population and inclusion process

Of 380 responses, 179 questionnaires were fully completed (47%) and 177 questionnaires were included in the analysis (paramedic field instructors *n* = 61; trainees *n* = 116). The main reason for exclusion was incomplete questionnaires (*n* = 201) (see Fig. 1). Due to variable-specific missingness, the number of observations among trainees varied slightly across analyses (*n* = 114–116).

67% of the sample came from two major fire departments (Berlin Fire Department, Hamburg Fire Department); the remaining participants were distributed across 11 of 16 German federal states (see Table 2). Table 3 shows baseline characteristics of the sample groups.

**Table 2:** Distribution of participants by organisation or federal stat**e**

Organisation / federal state	*n* (%)
Berlin Fire Department	63 (35.6%)
Hamburg Fire Department	56 (31.6%)
Schleswig-Holstein	12 (6.8%)
Lower Saxony	10 (5.6%)
North Rhine-Westphalia	9 (5.1%)
Bavaria	8 (4.5%)

Total sample *n* = 177. Only categories with n above seven are displayed

**Table 3:** Sample baseline characteristics of instructors and trainees

Variable	Instructors (*n* = 61)	Trainees (*n* = 116)
Work experience in EMS (years)		
Mean	14.8	–
Minimum	4	–
Maximum	38	–
Year of training	–	
Year 1	–	26 (23.2%)
Year 2	–	48 (42.9%)
Year 3	–	38 (33.9%)
Missing	–	4
Prior knowledge / training		
No specific prior knowledge	4 (6.6%)	26 (22.4%)
Human factors	39 (63.9%)	34 (29.3%)
Debriefing	46 (75.4%)	48 (41.4%)
Reflective practice	23 (37.7%)	16 (13.8%)
Crew/Team Resource Management	53 (86.9%)	86 (74.1%)

### Descriptive results

**Fig. 2:**
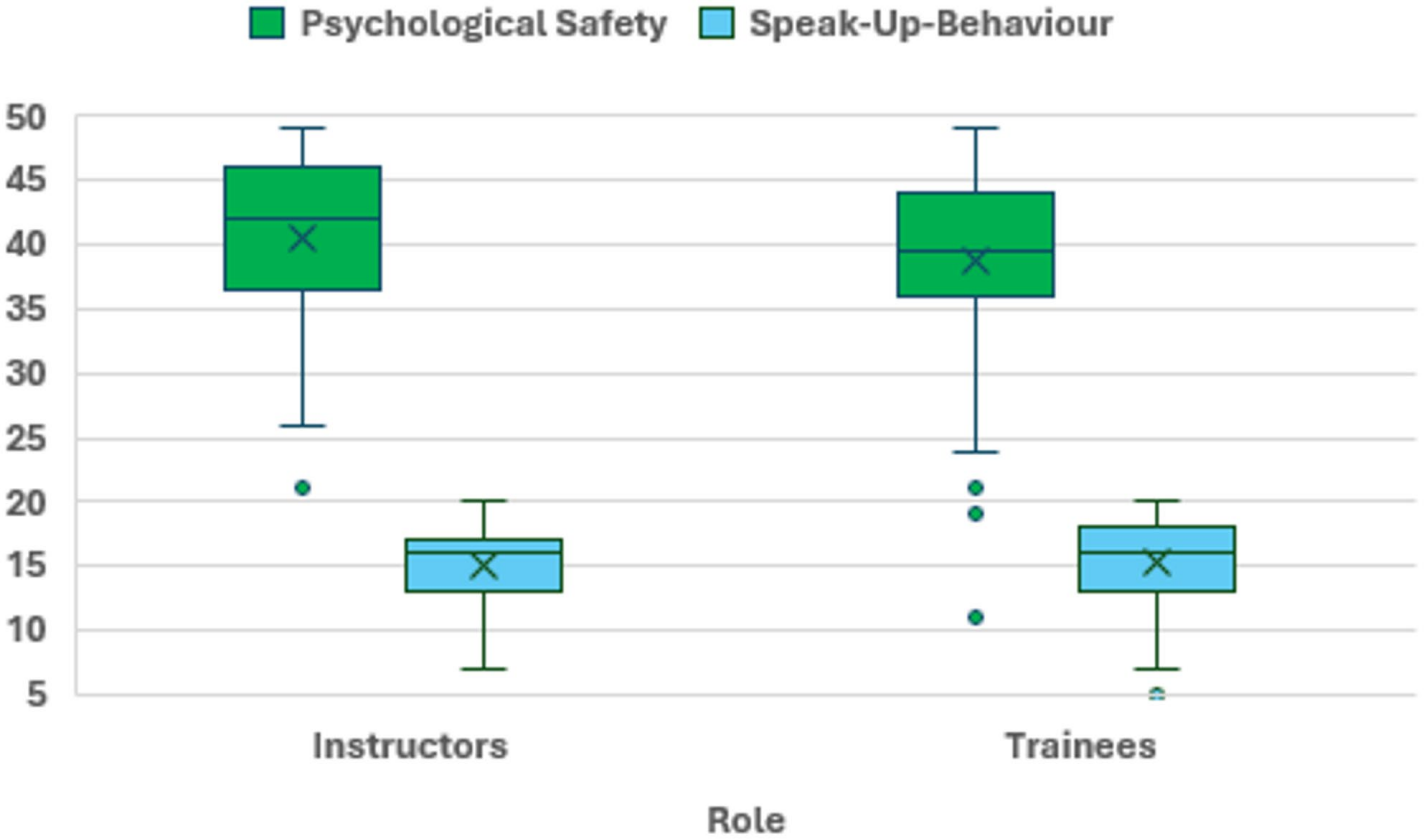
Boxplots of psychological safety and speak-up behaviour across roles. Levels of psychological safety and speak-up behaviour by group, in points. Minimum and maximum possible scores: psychological safety (Min. 7, Max. 49), speak-up behaviour (Min. 4, Max. 20)

Psychological safety scores were Mean (M) = 38.8 points (Standard deviation (SD) = 7.03 [Min. 11; Max. 49]) for trainees and M = 40.4 points (SD = 6.8 [Min. 21; Max. 49]) for paramedic field instructors. The speak-up scores differed only slightly (trainees M = 15.3 points; SD = 3.2 [Min. 5; Max. 20] vs. paramedic field instructors M = 15.2 points; SD = 2.8 [Min. 7; Max. 20]) (see Fig. 2).

**Fig. 3:**
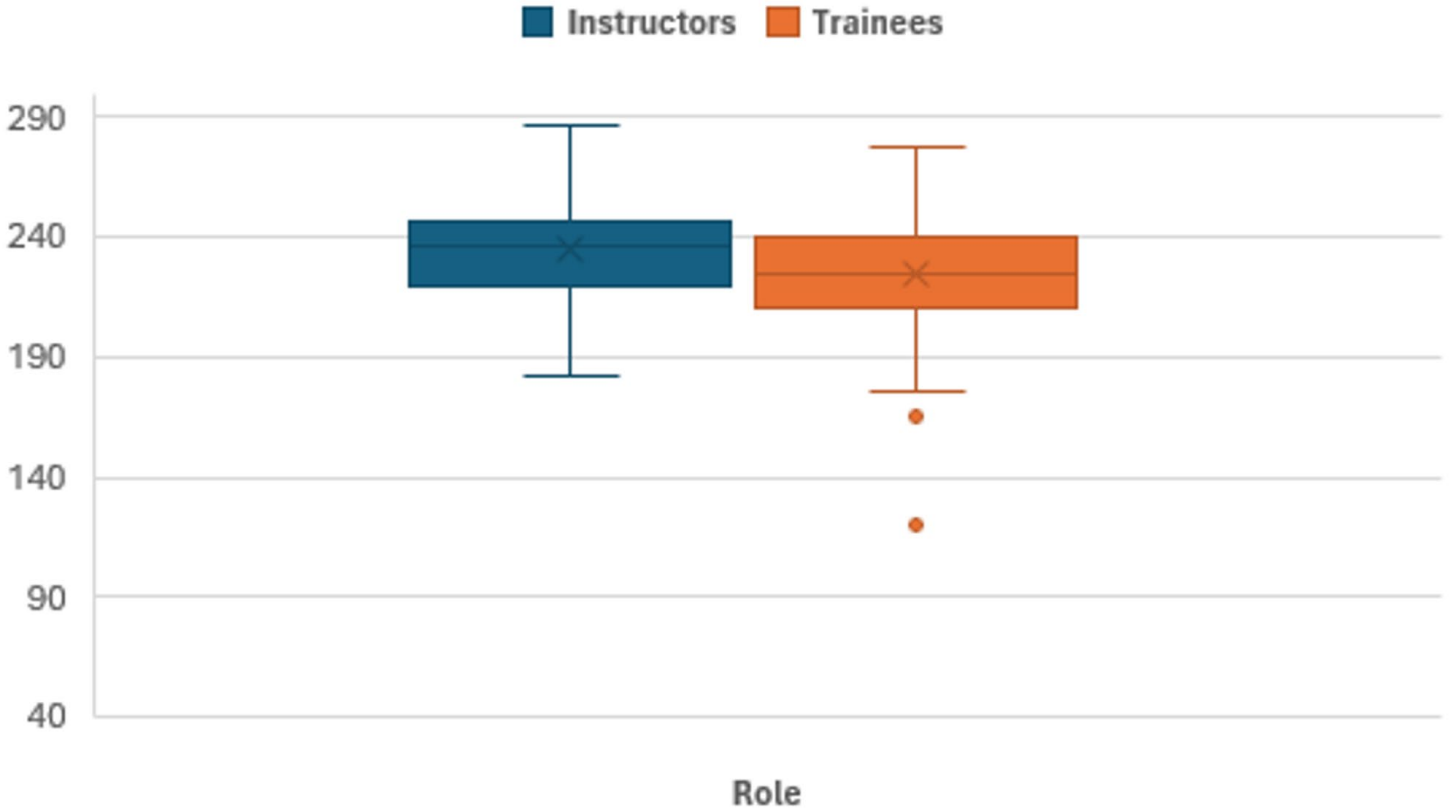
Boxplots of reflective competence across roles. Levels of self-reflection competence by group, in points. Minimum and maximum scores: Self-reflection competence (Min. 42, Max. 294).

Figure 3 shows the scores for self-reflection competence. The total self-reflection competence score was higher among paramedic field instructors (M = 234.5 points; SD = 23.7 [Min. 182; Max. 286]) than among trainees (M = 224.5 points, SD = 25.2 [Min. 120; Max. 278]).

**Fig. 4:**
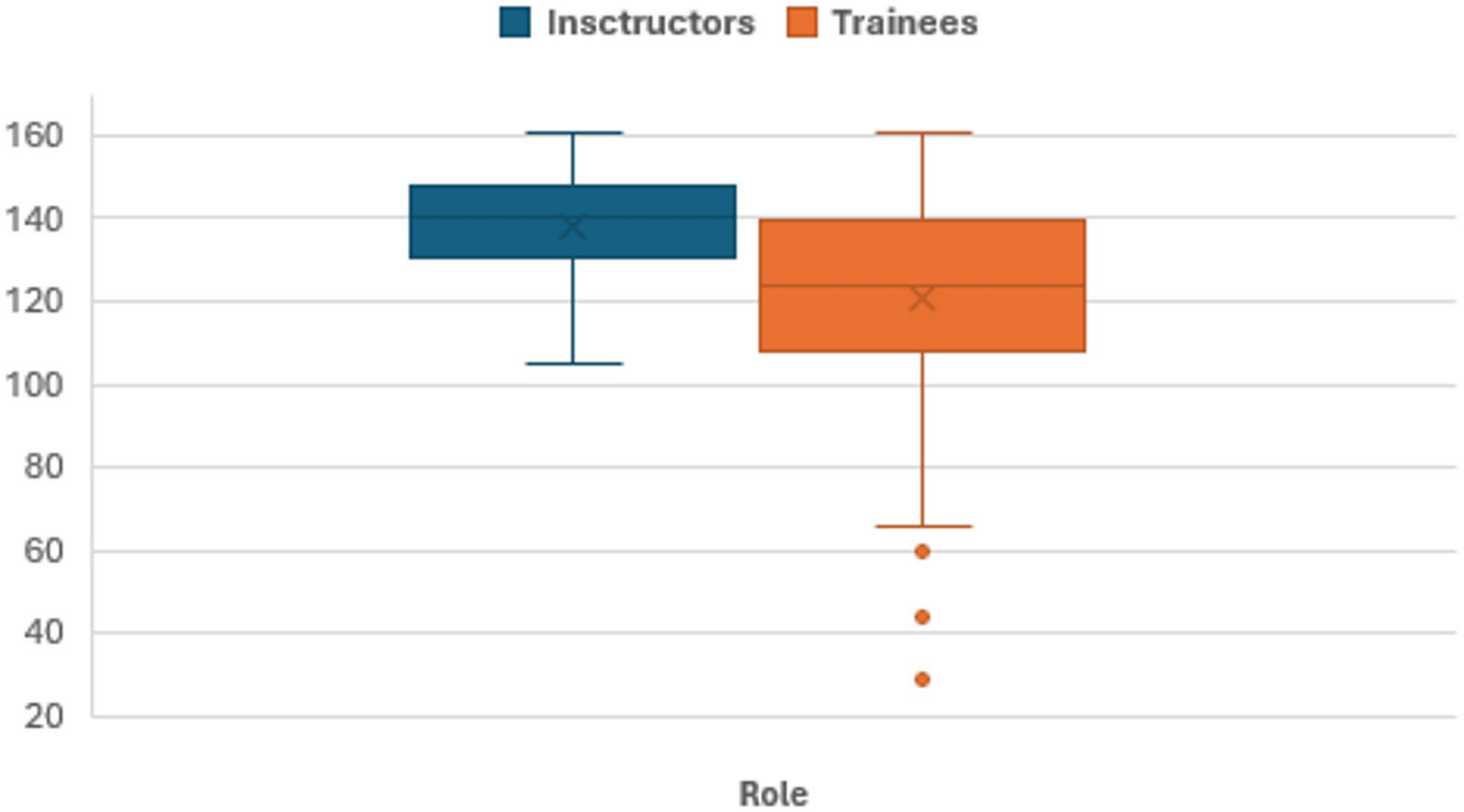
Boxplot of perceived debriefing quality across roles. Scores for perceived debriefing quality by group. Minimum and maximum possible scores: Debriefing quality (Min. 23, Max. 161).

The overall debriefing quality was also rated higher by paramedic field instructors (M = 138 points; SD = 12.6 [Min. 105; Max. 161]) than by trainees (*n* = 114; M = 121 points; 24.7 [Min. 29; Max. 161]), but the range was wider among trainees (see Fig. 4). It should be noted that the data do not refer to identical, jointly evaluated debriefing, but rather provide an exploratory, group-specific insight.

The Mann–Whitney U test indicated significant differences across roles in debriefing quality (*p* < .001) and reflection competence (*p* < .05), whereas no significant differences were observed for psychological safety and speak-up behaviour.

### Psychometric properties of the adapted DASH

Psychometric properties of the adapted DASH were analyzed separately for trainees and instructors. Internal consistency of the adapted DASH was high for both groups. For the overall scale, Cronbach’s α was 0.97 (*n* = 72) among trainees and 0.92 (*n* = 47) among instructors.

Subscale reliabilities differed between groups. Among trainees, Cronbach’s α ranged from 0.82 to 0.91 across subscales. Among instructors, subscale reliabilities were more variable, ranging from 0.56 to 0.82, with lower values observed particularly for the subscales “Effective learning atmosphere” (α = 0.60) and “Analysis of action” (α = 0.56).

In addition, the medians of corrected item–total and item–subscale correlations were examined (see Table 4). Item–total correlations were in an acceptable range across subscales in both groups. Item–subscale correlations were more variable and partially lower among instructors, whereas item–subscale correlations among trainees were consistently higher.

Inter-item correlations among trainees ranged from *r* = .20 to *r* = .86. Instructors showed a broader and more heterogeneous pattern of inter-item correlations (*r* = − .01 to *r* = .70), including several low and near-zero correlations. Examination of the inter-item correlation matrices revealed substantial correlations not only within but also between subscales, particularly among trainees. For example, correlations between items from “Analysis of action” and “Future orientation” reached up to *r* = .85, and correlations between “Structure” and “Reflection” items were frequently above *r* = .60.

Overall, the adapted DASH showed high internal consistency in both groups, with greater variability in subscale reliabilities and inter-item correlations among instructors and higher overall correlation patterns among trainees.

**Table 4:** Psychometric properties of the adapted DASH by group

	Subscales	Items (*n*)	Cronbachs α	Mean	Standard Deviation	Median of corrected Item-Total-Correlation [Range]	Median of corrected Item-Subscale-Correlation [Range]
Paramedic Field Instructors	**Effective learning atmosphere**	5	0.596 (*n* = 57)	31.05	2.850	0.522 [0.312–0.602)	0.457 [0.201–0.587]
	**Structure**	5	0.824 (*n* = 57)	29.72	3.740	0.643 [0.518–0.783]	0.623 [0.455–0.751]
	**Reflective discussion**	6	0.752 (*n* = 50)	35.24	4.565	0.583 (0.470–0.656]	0.509 [0.453–0.575]
	**Analysis of Action**	3	0.557 (*n* = 56)	17.68	2.064	0.513 [0.430–0.615]	0.375 [0.354–0.386]
	**Future Orientation**	4	0.813 (*n* = 57)	24.40	2.576	0.650 [0.541–0.736]	0.651 [0.504–0.752]
	**DASH-Scale-Intructors**	23	0.922 (*n* = 47)	138.72	13.527	-	-
Trainees	**Effective learning atmosphere**	5	0.822 (*n* = 91)	27.66	5.510	0.691 [0.560–0.747]	0.712 [0.338–0.729]
	**Structure**	5	0.902 (*n* = 99)	25.87	6.703	0.818 [0.806–0.857]	0.772 [0.674–0.807]
	**Reflective discussion**	6	0.855 (*n* = 80)	30.21	7.449	0.708 [0.514–0.765]	0.647 [0.550–0.736]
	**Analysis of Action**	3	0.901 (*n* = 101)	15.73	4.195	0.829 [0.818–0.882]	0.793 [0.782–0.839]
	**Future Orientation**	4	0.905 (*n* = 108)	22.26	4.951	0.824 [0.760–0.854]	0.796 [0.728–0.829]
	**DASH-Scale-Trainees**	23	0.969 (*n* = 72)	122.58	26.046	-	-

Psychometric properties of the adapted DASH by group.Cronbach’s α is reported for each subscale and for the overall scale. Values for corrected item–total and item–subscale correlations are presented as medians with ranges (minimum–maximum) across items. Item–total correlations refer to correlations between each item and the total score of the respective scale excluding the item itself. Item–subscale correlations refer to correlations between each item and the sum score of its corresponding subscale excluding the item. Analyses were conducted separately for trainees and instructors. Sample sizes (*n*) vary across subscales due to missing data

### Main correlations

The correlations of the main scales by group are presented in Table 5. In the group of paramedic field instructors, the strongest correlation was found between self-reflection competence and self-rated debriefing quality (*r* = .67; *p* < .001). Debriefing quality also correlated with psychological safety (*r* = .396; *p* < .002) and speak-up (*r* = .295; *p* < .036). Psychological safety and speak-up were significantly associated (*r* = .623; *p* < .001). The correlations between self-reflection competence and speak-up (*r* = .269; *p* < .036) and psychological safety (*r* = .229; n. s.) were weaker.

Among trainees, the strongest correlations were observed between psychological safety and debriefing quality (*r* = .614; *p* < .001) as well as between speak-up and debriefing quality (*r* = .655; *p* < .001). Self-reflection competence correlated moderately with debriefing quality (*r* = .335; *p* < .001). Psychological safety and speak-up were also significantly correlated (*r* = .596; *p* < .001).

**Table 5:** Correlations of the main scales by group

		1 Self-reflection competence	2 Psychological Safety	3 Speak-up
**Paramedic fieldinstructors (** ***n*** **= 61)**	**1 Self-reflection competence**	1		
	**2 Psychological Safety**	0.229	1	
	**3 Speak-up**	0.269*	0.623**	1
	**4 Quality of debriefing**	0.670**	0.396**	0.295*
**Paramedic trainees**	**1 Self-reflection competence**	1		
	**2 Psychological Safety**	0.442** (*n* = 116)	1	
	**3 Speak-up**	0.298** (*n* = 116)	0.596** (*n* = 116)	1
	**4 Quality of debriefing**	0.335** (*n* = 114)	0.614** (*n* = 114)	0.655** (*n* = 114)

Significant correlations are highlighted in bold. Significance levels: * = significant (*p* < .05); ** = significant (*p* < .01)

### Exploratory debriefing quality subscale correlations

In the (exploratory) subdimensions, all aspects of debriefing quality among paramedic field instructors correlated significantly with self-reflection competence (weakest correlation: “Analysis of Action,” *r* = .45; strongest correlation: “Future Orientation,” *r* = .647; *p* < .001 in each case). Among trainees, the strongest correlation was observed between “Effective learning atmosphere” in the debriefing and self-reflection competence (*r* = .43; *p* < .001), while the weakest was between “Structure” and self-reflection competence (*r* = .257; *p* < .006) (see Table 6).

Among paramedic field instructors, the strongest correlation regarding the relationships between psychological safety and specific aspects of debriefing quality was found for “Structure” (*r* = .418; *p* < .001), and the weakest for “Reflective discussion” (*r* = .255; *p* < .047). For trainees, the correlations were stronger overall; the weakest correlation was also found for “Reflective discussion” (*r* = .502; *p* < .001), and the strongest for “Effective learning atmosphere” (*r* = .639; *p* < .001) (see Table 6).

**Table 6:** Correlations between the debriefing quality subscales and reflective competence and psychological safety by group

		Quality of debriefing	Effective learning atmosphere	Structure	Reflective discussion	Analysis of Action	Future Orientation
**Paramedic field instructors (** ***n*** ** = 61)**	**Self-reflection competence**	0.670**	0.492**	0.614**	0.530**	0.450**	0.647**
	**Psychological Safety**	0.396**	0.319*	0.418**	0.255*	0.344**	0.306*
**Paramedic trainees**	**Self-reflection competence**	0.335** (*n* = 114)	0.430** (*n* = 114)	0.257** (*n* = 114)	0.262** (*n* = 114)	0.260** (*n* = 114)	0.289** (*n* = 116)
	**Psychological Safety**	0.614** (*n* = 114)	0.639** (*n* = 114)	0.535** (*n* = 114)	0.502** (*n* = 114)	0.525** (*n* = 114)	0.551** (*n* = 114)

Significant correlations are highlighted in bold. Significance levels: * = significant (*p* < .05); ** = significant (*p* < .01)

### Exploratory self-reflection competence subscale correlations

The correlations between the subdimensions of reflective competence and debriefing quality are presented in Table 7. Among paramedic field instructors, goal setting (*r* = .580; *p* < .001) and action optimization (*r* = .565; *p* < .001) showed particularly strong correlations with perceived debriefing quality. Among trainees, the strongest association was observed for descriptive reflection (*r* = .362; *p* < .001), whereas other subdimensions showed weaker or non-significant correlations.

In the exploratory subscale analysis among paramedic field instructors, a significant correlation was found between psychological safety and specific components of self-reflection competence only for “Reflection success” (*r* = .314; *p* < .014). Among trainees, “Descriptive reflection” in particular correlated significantly with psychological safety (*r* = .541; *p* < .001); other subdomains showed significant correlations ranging from *r* = .226 to *r* = .378.

**Table 7:** Correlations between the reflection competence subscales and debriefing quality by group

	Paramedic field instructors (*n* = 61)	Paramedic trainees (*n* = 114)
	Quality of debriefing	
**Self-reflection competence**	0.670**	0.335**
**Descriptive reflection**	0.278*	0.362**
**Cause analysis**	0.426**	0.062
**Goal setting**	0.580**	0.357**
**Action optimization**	0.565**	0.324**
**Willingness to reflect**	0.546**	0.166
**Reflection success**	0.488**	0.319**

Significant correlations are highlighted in bold. Significance levels: * = significant (*p* < .05); ** = significant (*p* < .01)

### Exploratory analyses of potential contextual factors

Exploratory analyses examined potential contextual factors associated with the main constructs. Among instructors, psychological safety was significantly associated with team familiarity, with lower familiarity (< 10 shared shifts) showing a negative association and higher familiarity (> 10 shared shifts) showing a positive association. In addition, prior knowledge was positively associated with psychological safety and speak-up behaviour in this group. No consistent or significant associations were observed among trainees, and experience (years of professional experience or training level) was not significantly related to the main variables in either group.

## Discussion

This study examined the relationships between debriefing quality, self-reflection competence, and psychological safety in emergency medical services training, focusing on both paramedic trainees and field instructors. The findings highlight both shared patterns and role-specific differences in how these constructs are interrelated.

### Psychometric consideration of adapted DASH

The psychometric findings of the adapted DASH provide important context for interpreting the observed associations. While internal consistency was high, the substantial overlap between subscales suggests that participants may have perceived debriefing quality as a more global construct rather than as clearly separable dimensions. This pattern was observed in both groups, although it appeared more pronounced among trainees. This interpretation is further supported by the observed pattern of item–subscale correlations, which were more variable and partially lower among instructors, indicating a less clearly differentiated subscale structure. However, alternative explanations such as reduced scale coherence or context-related variability cannot be excluded. This is conceptually plausible, as core elements of debriefing - such as structure, reflection, and future-oriented learning - are inherently interconnected processes.

Differences between trainees and instructors further support this interpretation. Trainees showed greater variability in their overall ratings but stronger inter-item correlations, indicating a more global evaluation pattern characterized by high internal consistency alongside substantial redundancy. In contrast, instructors demonstrated less variability in overall ratings but weaker inter-item correlations, suggesting a less consistent and less clearly structured evaluation of specific debriefing components. These differences may reflect varying levels of experience and familiarity with debriefing practices.

These findings should be interpreted in light of the adaptation of the DASH. The instrument was originally developed as an observational tool for structured simulation-based debriefings, whereas the present study applied a self-report format in a real-world setting. In this context, debriefings are typically less standardized and more dependent on situational factors. Under these conditions, participants may form more holistic impressions rather than differentiating between specific components, which may limit the instrument’s ability to capture distinct sub-dimensions.

In addition, the use of a self-report format may introduce systematic biases, as participants’ evaluations may be influenced by individual perceptions, social desirability, or their overall impression of the debriefing rather than specific observable behaviours. This raises questions regarding construct validity, as the instrument may capture a more global evaluation of the debriefing experience rather than distinct underlying dimensions as originally conceptualized.

In summary, these findings suggest that the adapted DASH is suitable for assessing overall perceptions of debriefing quality in real-world settings, while its dimensional structure may not fully translate to this context. In particular, the findings suggest that while internal consistency is high, evidence for discriminant validity between subdimensions is limited. Further research should investigate how debriefing processes can be measured in practice and how different components contribute to psychological safety, reflection, and speaking up.

### Group differences and variability

Although the groups differ substantially in terms of professional experience, the mean levels of self-reflection competence were relatively similar, despite a statistically significant difference. This may suggest that factors other than experience contribute to the development of this construct. However, no direct conclusions regarding the influence of work experience can be drawn based on the present data. Across all constructs examined, mean scores were generally comparable between trainees and paramedic field instructors. However, a wider range of responses was observed among trainees, suggesting greater variability within this group. This may reflect more heterogeneous experiences. The underlying causes of this variability cannot be determined based on the available data.

### Role-specific patterns: instructors vs. trainees

The relationships between the examined constructs differed between paramedic field instructors and trainees, suggesting role-specific patterns in how debriefing is experienced and interpreted.

### Paramedic field instructors

The strong association between self-reflection competence and debriefing quality for the paramedic field instructors emphasizes the role of the facilitator. This finding appears to underscores the close connection between one’s own capacity for reflection and the design and perception of structured debriefing. The strong association between self-reflection competence and perceived debriefing quality among instructors suggests that facilitators’ reflective capacity may be relevant to how debriefing is conducted and experienced. For instance, in a qualitative study on the reflective competence of paramedic students following simulations, no superiority was found between instructor-led vs. participant-led trainings [29]. A meta-analysis also failed to establish a clear connection. Although the analysis found that facilitator-led debriefing was more effective, the findings were limited due to the evidence was based on very few studies without facilitators-led debriefing [2]. Kolbe et al. (2023) found a positive correlation between communication patterns involving open-ended questions from the facilitator and the sharing of mental models as part of the reflection process in the form of participants’ responses [30]. Here, too, a certain level of reflective competence can be assumed to be necessary for this type of communication. One possible explanation for this may be that reflective competence influences the nature of the questions asked, the structure of the discussion, and the promotion of shared analytical processes. Nevertheless, targeted professional development for facilitators is both a prerequisite for long-term implementation [4, 8], so that the findings can be incorporated into these requirements and complement the existing competencies of facilitators [31].

The exploratory subscale analyses emphasize the need to design debriefing as a positive, self-directed learning process: Significantly correlated dimensions describe, in particular, the processing of experiences with a view to future action and suggests to highlight the function of debriefing as a tool for deriving actionable insights. This corresponds to theoretical concepts of experiential learning, which understand reflection as the link between experience and future action [9–11]. Furthermore, the findings may underscore the need to consider volitional components in the overall assessment of reflective competence [13], which is clearly linked to positive experiences and a strong practical relevance. This makes it all the more important to create positive experiences for trainees in order to provide them with a solid foundation for experience-based lifelong learning.

### Paramedic trainees

For trainees, psychological safety appears to be closely linked to the opportunity to actively participate and to experience reflection as conducive to learning. This finding is consistent with the literature, which describes psychological safety as a key prerequisite for learning, participation, and the reporting of errors [8, 10, 11, 32].

This is also confirmed by the exploratory subscale analyses: here, the perceived learning atmosphere in particular was closely linked to reflective competence. For this group, it seems that the quality of the framework in which reflection can take place is more decisive than the goal-oriented derivation of action strategies. A constructive, non-judgmental, and learning-oriented atmosphere is thus closely linked to the effects of the reflection processes. An additional aspect of this study concerns the importance of the descriptive level in the debriefing process for trainees. These findings suggest that, in particular, the opportunity to first describe one’s own perceptions and experience of a situation could be relevant to the perception of psychological safety. The opportunity to describe observations, thoughts, and decision-making processes initially at a descriptive level, before a deeper analysis or evaluation takes place, may facilitate participation and help promote psychological safety in the reflection process. This underscores the value of a debriefing process that first provides space for the presentation of individual perceptions before interpretive or evaluative steps are taken [1, 14, 20].

These findings suggest that debriefing may be experienced differently depending on the role, with a stronger emphasis on facilitation and reflective structuring for instructors, and on psychological safety and participatory learning conditions for trainees.

### Psychological safety and interpersonal risk

In both groups, there was a correlation between psychological safety and speak-up behaviour. This supports findings that describe psychological safety as an important predictor of speaking up about uncertainties or observations, and points to debriefing as a potential framework for fostering open communication [11, 19, 32, 33]. However, it seems that not all topics can be addressed with the same openness: issues related to patient safety appear to be easier to address than interpersonal or team-related conflicts, which may involve higher perceived interpersonal risk [11]. Beyond the observed associations, the findings may also be interpreted through the lens of interpersonal risk. Situations involving evaluation, uncertainty, or potential error inherently carry the risk of negative judgment, particularly in hierarchical training environments. In this context, psychological safety can be understood as a condition that may reduce the perceived risks associated with participation in reflection and communication processes [3]. The close associations observed between debriefing quality, psychological safety, and speak-up behaviour support the notion that these constructs are closely intertwined in practice.

In the context of emergency medical services training, these dynamics may be further influenced by hierarchical structures. Debriefings often take place within a structured power dynamic, where instructors facilitate the process and trainees occupy a formally subordinate learning role. Previous research suggests that hierarchical differences can affect psychological safety, with higher-status individuals often reporting greater perceived safety [34, 35]. The strong link between psychological safety and the quality of debriefing, as well as trainees’ reflective competence, suggests that a safe framework is particularly important under conditions of hierarchical asymmetry.

The exploratory analyses of contextual factors further support these findings. For paramedic field instructors, psychological safety appeared to be more strongly associated with structural and contextual conditions, such as familiarity within the team and prior knowledge. In this group, psychological safety also seemed to depend on the presence of a clear and structured approach to debriefing. This finding was also observed in a qualitative study conducted in a clinical setting, in which a standardized, formal communication process was associated with psychological safety [36]. In an implementation study, the instructors also reported that using a structured approach for preclinical debriefing helped facilitate effective facilitation [37]. The results of a meta-analysis were inconclusive in this regard, as very few studies included debriefing without a structured format. Although the evidence remains limited, these findings may indicate that structure plays an important role in enabling psychologically safe debriefings for instructors [2]. 

A focus on learning, familiarity and trust within the team, clarity of roles, and a standardized communication process have been shown to promote psychological safety in other settings and could serve as practical approaches [11, 20, 32, 36]. These findings further emphasize that for instructors, psychological safety may be more strongly rooted in stable contextual conditions, while for trainees it appears to be more situationally constructed within the debriefing itself. Although potential contextual factors were explored, these variables were not included in multivariable models. Therefore, potential confounding effects cannot be ruled out, and the observed associations should be interpreted with caution.

Taken together, these findings support the notion that psychological safety, communication, and reflection are closely intertwined in practice, and that debriefing may provide a structured setting in which interpersonal risk can be reduced and open communication facilitated.

### Implications for practice and training

The findings of this study may have practical implications for the design and implementation of debriefings in emergency medical services training. In particular, they highlight the importance of combining structured reflection with a psychologically safe learning environment.

For paramedic field instructors, the results suggest that facilitation skills and reflective competence may be relevant for conducting effective debriefings. Training programs may therefore benefit from focusing not only on methodological aspects of debriefing, but also on the development of reflective and communicative competencies. In addition, the findings indicate that the use of structured approaches may support facilitators in guiding reflection processes and in creating psychologically safe conditions.

For trainees, the findings emphasize the importance of a supportive and non-judgmental learning environment. Providing opportunities for active participation, particularly through initial descriptive reflection before evaluative discussion, may facilitate engagement and promote psychological safety.

These findings suggest that debriefing should be understood as a deliberately designed learning space that integrates structure, facilitation, and interpersonal dynamics. Embedding debriefing in training curricula and organizational practice may therefore support the development of reflective competence, communication, and team-based learning in emergency medical services.

### Limitations

The results must be interpreted in light of several limitations. Because the majority of participants were affiliated with two organizations, the sample is only partially representative, meaning that organization-specific influences cannot be ruled out. The cross-sectional design does not allow for conclusions regarding causality or temporal developments in the observed relationships. The target sample size for the group of paramedic field instructors was not reached, meaning that the subgroup analyses are underpowered and smaller effects are not detectable. Furthermore, all data are based on self-reports, which means that distortions due to social desirability or individual perception are possible. The measurement instruments used here have not yet been validated for the emergency medical services context, which may limit the interpretability of the observed associations, so the results are exploratory in nature. Finally, selection bias due to voluntary participation cannot be ruled out. The low variance in the construct scores may indicate this. A potential nonresponse bias cannot be excluded, given that only 47% of questionnaires were fully completed. This may have resulted in a selective sample of participants with a greater interest in debriefing or more positive perceptions. This could partly explain the overall high scores observed across variables and the comparatively limited variability in some measures.

The use of self-report measures represents an important limitation of the present study. Participants’ ratings may be influenced by subjective perceptions, social desirability, or a tendency to overestimate one’s own competence, particularly among learners. This may affect the accuracy of reported levels of reflection, psychological safety, or speak-up behaviour. In addition, all variables were assessed using the same method and source, which may introduce common method bias and artificially inflate observed associations. Previous research has shown that relationships between constructs such as psychological safety and related outcomes may be overestimated when measured using single-source self-report data [32]. Therefore, the observed associations should be interpreted with caution. Future studies may benefit from combining self-report measures with observational or multi-source data to obtain a more comprehensive assessment of debriefing processes.

Although learner level (e.g., year of training) and professional experience were explored as potential contextual factors, no consistent associations with the main constructs were observed in the present sample. However, these findings should be interpreted with caution. The study was not specifically powered to detect subgroup differences or interaction effects, and more complex relationships between experience, psychological safety, reflection, and speak-up behaviour may therefore have remained undetected. Previous research suggests that differences in experience may meaningfully shape how individuals engage in reflection and perceive psychologically safe environments. Future studies should therefore examine these relationships using designs that allow for more detailed subgroup or interaction analyses.

Furthermore, trainee and instructor ratings were not linked to identical debriefing events, which limits direct comparability between groups. The findings should also be interpreted in light of the context-sensitive nature of debriefing in real-world settings. In practice, debriefings are rarely conducted in a standardized manner but are dynamically adapted to factors such as learner level, situational complexity, and the emotional tone of the event.

These factors may influence both the structure of the debriefing and the way participants engage in reflection and communication. For example, more complex or emotionally charged situations may require greater emphasis on psychological safety. Although these contextual factors were not systematically examined in the present study, their potential influence should be considered when interpreting the observed relationships. Future research may benefit from explicitly incorporating such situational and contextual dimensions to better understand how debriefing processes unfold in practice.

### Open questions and further research needs

Future studies should employ longitudinal designs to examine developmental trajectories and potential causal relationships between debriefing quality, psychological safety, and reflective competence. Cross-organizational studies could also clarify the extent to which structural conditions and hierarchical structures influence the results and whether they are transferable to other emergency service settings.

## Conclusions

These findings suggest that debriefing in emergency medical service training is associated with both reflective capacity and psychologically safe communication, with differing patterns for trainees and field instructors. In the emergency medical services context, debriefing maybe understood not merely as a review of incidents, but as a purposefully designed space for learning and communication. Its effectiveness may stems from the interplay of structured reflection, facilitation skills, and an exchange that is perceived as safe. The requirements for conducting a high-quality debriefing may vary depending on the role (participant vs. facilitator).

In practice, this this highlights the potential valueembedding debriefing in training and organizational culture. Paramedic field instructors play a key role in this process, as their ability to guide reflection processes and create a learning-friendly environment appears to belinked to the perceived quality of the debriefing. When debriefing is implemented appropriately, it may foster an open culture of communication and, in the long term, contribute to strengthening teamwork, confidence in decision-making, and the quality of care in the preclinical setting.

## Data Availability

The datasets used and analysed during the current study are available from the corresponding author on reasonable request.
